# The In Vitro Inhibitory Activity of Pacaya Palm Rachis versus Dipeptidyl Peptidase-IV, Angiotensin-Converting Enzyme, α-Glucosidase and α-Amylase

**DOI:** 10.3390/plants13030400

**Published:** 2024-01-29

**Authors:** Aurea Bernardino-Nicanor, Stephanie Fernández-Avalos, José Mayolo Simitrio Juárez-Goiz, José Luis Montañez-Soto, Leopoldo González-Cruz

**Affiliations:** 1 Tecnológico Nacional de México/ IT de Celaya, Antonio-García Cubas Pte #600 Esq. Av. Tecnológico, Celaya, Guanajuato C.P. 38010, Mexico; aurea.bernardino@itcelaya.edu.mx (A.B.-N.); m2003064@itcelaya.edu.mx (S.F.-A.); mayolo.juarez@itcelaya.edu.mx (J.M.S.J.-G.); 2 Centro Interdisciplinario de Investigación para el Desarrollo Integral Regional del, Instituto Politécnico Nacional, Jiquilpan, Michoacan C.P. 59510, Mexico; j.montanez@ipn.mx

**Keywords:** *Chamaedorea tepejilote* Liebm, rachis, thermal treatment, antidiabetic, antihypertensive

## Abstract

The pacaya palm (*Chamaedorea tepejilote* Liebm) is an important food that is commonly consumed in Mexico and Central America due to its nutritive value. It is also used as a nutraceutical food against some chronic diseases, such as hypertension and hyperglycemia. However, few reports have indicated its possible potential. For this reason, the goal of this research was to evaluate the effects of the enzymatic activity of the pacaya palm inflorescence rachis on both hypertension and hyperglycemia and the effects of thermal treatments on the enzymatic activity. The enzymatic inhibition of ACE (angiotensin-converting enzyme), DPP-IV (dipeptidyl peptidase-IV), α-glucosidase and α-amylase were evaluated, all with powder extracts of pacaya palm inflorescences rachis. The results indicated that thermally treated rachis showed increased enzymatic inhibitory activity against α-amylase and DPP-IV. However, all rachis, both with and without thermal treatment, showed low- or no enzymatic activity against α-glucosidase and ACE. Apparently, the mechanism of action of the antidiabetic effect of rachis is mediated by the inhibition of α-amylase and DPP-IV and does not contribute with a significant effect on enzymes involved in the hypertension mechanism. Finally, the properties of the extract were modified via the extraction method and the temperature tested.

## 1. Introduction

Chronic diseases such as diabetes and hypertension are a major cause of morbidity and mortality worldwide [1], with diabetes mellitus being a disease characterized by chronic hyperglycemia caused by changes in insulin secretion [2]. Some mechanisms of action of the drugs used in the treatment of diabetes mellitus involve the inhibition of some enzymes such as α-amylase and α-glucosidase, which are responsible for the hydrolysis of complex carbohydrates to simple carbohydrates such as glucose [3]. For this reason, it has been documented that inhibitors of α-amylase and α-glucosidase reduce the absorption of dietary carbohydrates, suppress postprandial hyperglycemia and could be an alternative in the treatment of patients with diabetes mellitus [4]. Recent studies have shown that iminosugars with conformational changes in the side chain have a high therapeutic efficacy [5]. An example of these compounds is miglitol, an antidiabetic agent that focuses on the inhibition of the enzyme α-glucosidase [6]. In addition, structurally modified carbohydrates have been reported to act as mimetics of natural carbohydrates and exhibit various biological activities [7]. One example are annulated sugars such as peltalosa, which is found in *Psacalium peltatum* and exhibits antidiabetic activity [8].

On the other hand, inhibitors of the DPP-IV enzyme are used in the treatment of diabetes because they increase the half-life of incretins [9], which are peptide hormones that stimulate more than 50% of insulin secretion, especially the hormone glucagon peptide (GLP-1) [10]. By inhibiting the DPP-IV enzyme, the half-life of incretins is increased and, for this reason, the effect of incretins is also enhanced and greater stimulation of β-pancreatic cells is produced [11].

Another common chronic disease is hypertension, which could affect 29% of the world’s adult population by 2025 [12]. Considering that hypertension is a progressive disease that triggers other chronic diseases such as cardiovascular disease and stroke [13], it is important to study the renin–angiotensin system, which regulates blood pressure as follows: renin activates angiotensinogen to produce angiotensin I, which is subsequently converted to angiotensin II by the action of the ACE enzyme. For this reason, ACE inhibitors are a therapeutic target against high blood pressure that reduce the concentration of angiotensin II and thus lower blood pressure [14].

Due to the therapeutic efficacy of enzyme inhibitors, the search for plants with a certain nutraceutical activity is a priority, e.g., *Acacia nilotica*, *Acacia Senegal*, *Withania somnifera*, *Ficus hispida*, *Hibiscus sabdriffa*, *Ginkgo biloba*, *Ocimum sanctum* and *Allium sativum* have shown antihypertensive and diuretic properties, and ACE enzyme inhibitory activity has also been reported in “in vitro” and in “in vivo” tests [15]. On the other hand, plants such as *Abelmoschus moschatus*, *Bidens pilosa*, *Acacia arabica* and *Ferula assafoetida* L. have been reported to have antidiabetic properties [16,17,18,19].

*Chamaedorea tepejilote* Liebm (pacaya palm) is a plant that is eaten in Central America and Mexico and is also used in traditional medicine as a supportive therapy for respiratory diseases such as coughs, bronchitis, pneumonia and colds [20]. Some studies (such as that carried out by Pérez et al. [21]) have shown that methanol extracts from pacaya palm have antitussive effects; meanwhile, Jiménez et al. [22] reported that pacaya palm has antimicrobial activity against *Mycobacterium tuberculosis*. In some regions of Mexico, the pacaya palm is used as a medicinal plant due to its antidiabetic effect. In addition, tepejilote inflorescences have been reported to lower postprandial blood glucose in normoglycemic mice without heat treatment [23], and male inflorescences of powdered tepejilote have also been shown to inhibit the enzyme DPP-IV [24]. On the other hand, people principally consume two parts of the pacaya palm inflorescences after thermal treatment: the rachis and rachilla. Some studies have reported that thermal treatment modifies the structures of some components, such as proteins [25], and changes the extractability of other components (e.g., an increase in the extractability of lutein and β-carotene in nopalitos was reported by Jaramillo et al. [26]). Additionally, other authors have indicated that thermal treatment improves the extractability of coumarins [27] and phenolic compounds [28], as well as impacting the stability and bioactivity of dietary flavonoids [29]. Heat treatment has been reported to alter not only the extractability of bioactive compounds, but also the biological activity of foods, such as by increasing antioxidant activity [30], enhancing cytotoxic activity against colon cancer cells [31] and increasing α-glucosidase inhibitory activity [32]. It has also been observed to positively alter antimicrobial activity [33].The beneficial effects of thermal treatment on diverse biological activity have been reported; however, other studies have also indicated that thermal treatment reduces the ability of some compounds to inhibit chronic diseases or biological activity. For example, an 8% reduction in the antioxidant activity of betalains following thermal treatment has been reported [34]. On the other hand, it has also been reported that the degree and duration of thermal processing are important factors for the preservation of components, such as phenolic compounds, and their antiproliferative activity [35]. In addition, it has been observed that the antihypertensive effect of onion disappears after even mild thermal treatment. For the above reasons, and also considering that both the rachis and rachilla of the pacaya palm inflorescences are commonly consumed after thermal treatment and that few studies have reported on the effects of the pacaya palm inflorescences on the enzymatic activity related to chronic diseases, such as diabetes and hypertension, the aim of this study was to determine the effects of thermal treatments on the enzymatic activities of samples of vacuum-packed rachis.

## 2. Results and Discussion

### 2.1. Effects of Extraction Solvent on Extract Yields

As shown in Table 1, all thermal treatments increased the extraction yield; however, a higher extraction percentage was obtained when water was used as an extraction solvent than when hexane was used. Treatment with steam at elevated pressure showed the highest extraction yield: about 0.7-fold and 1.3-fold for water and hexane, respectively, compared to the extract yields obtained from rachis without thermal treatment.

Other studies have also found differences between extraction yields when water and hexane were used as solvents, e.g., in a study on the *Datura metel* plant, a yield percentage of 78% was reported when water was used as a solvent, while 19.4% was obtained when hexane was used [36]. On the other hand, *Anthurium schlechtendalii* is a plant belonging to the *Araceae* family, and in one study, percentage yields of 18% (water) and 1.4% (hexane) were reported and, apparently, higher yields are obtained when water is used as a solvent due as the natural hydrophilic compounds predominate in the extracts of the plants studied [37].

According to our results, the thermal treatments improved the extraction of components from the rachis matrices. This was in concordance with the results reported by Esteves et al. [38], who indicated that the extractive contents increased in the first hours of thermal treatment, while Lambri et al. [39], reported that the application of high temperatures resulted in the same extraction yields of the anthocyanins. Other authors have also reported that steam treatment could increase the percentage yields of methanolic extracts from sweet potato flour three- or four-fold [40]. These results indicate that structural cellular damage occurs, as does the partial hydrolysis of biopolymers, thereby enhancing the percentage yields of components.

### 2.2. Modifications to the Total Phenolic Compound Content

In the rachis extracts, both aqueous and hexanoic, a significant increase (*p* < 0.05) in total phenolic content was observed after thermal treatment; however, as shown in Table 2, a more efficient extraction process was achieved when water was used as the extraction solvent. The effects of the thermal treatments were lower in the aqueous extracts than the hexane extracts; apparently the use of water as the extraction solvent or as an adjuvant in the thermal process enhanced the extractability of phenolic compounds. For this reason, in the hexane extracts, the total phenolic content was higher when a wet treatment was used (i.e., hydrothermal processing and steaming at an elevated pressure) than when a dry treatment (microwaving) was used.

It has been observed that various thermal treatments can modify the extractability of phenolic compounds. For example, in bitter gourd, a higher phenolic compound content was extracted following a roasting process of between 200 and 250 °C compared to that from fresh bitter gourd [41]. Additionally, our results were in concordance with those reported by Chumyam et al. [42], who used similar thermal treatment (boiling, steaming and microwaving) and found that thermally treated eggplant had a higher phenolic compound content than fresh eggplant.

The highest extractability of phenolic compounds was obtained when hexane was used as extraction solvent in hydrothermal processing and steam treatment at elevated pressure, which is due to the fact that a lot of moisture is retained in these treatments, facilitating the migration of the solvent. In addition, the solubilization of the pectic compounds in the lamella media increases the permeability of the cell walls [43], while the lower extraction of the phenolic compounds in the microwave-treated samples is due to the stiffness of the rachis cell walls. Some authors have pointed out that this phenomenon is caused by the crystallization of cellulose and hemicellulose during dry heat treatment, which reduces the migration of the solvent and the extraction of the phenolic components [44]. A study on the rachis of *Moringa oleifera* shows that the antioxidant activity could be due to the total content of polyphenols [45], which is consistent with the results obtained in this study. In addition, the presence of flavonoids has been reported in the *Aracaceae* family [46], to which *Chamaedorea tepejilote* Liebm belongs. For this reason, new studies are needed to identify the specific component responsible for the antioxidant and enzymatic activities.

### 2.3. Effects of Thermal Treatment on the α-Amylase Inhibition Activity of the Rachis

As shown in Table 3, at a concentration of 250 µg, the thermally treated samples (both powder and aqueous extract) showed higher α-amylase inhibitory activity than the samples without thermal treatment or acarbose (positive control); however, at a concentration of 500 µg, a similar patron was detected only in the aqueous extracts. A lower percentage inhibition of α-amylase was observed in the powder than in the aqueous extracts. Nevertheless, a lower α-amylase inhibitory activity was observed in the thermally treated powder samples than in the aqueous extracts at a dose of 500 µg, but the α-amylase inhibitory activity did not exceed the values obtained for the positive control (acarbose). Different results were obtained for the aqueous extracts.

A dose-dependent effect was observed among the aqueous extracts; however, it was more evident in samples that had undergone a wet treatment. The highest dose-dependent increase in enzymatic inhibition activity was observed in the rachis without thermal treatment.

Some other authors have also reported that thermal treatments increase α-amylase inhibition activity, e.g., the α-amylase inhibition activity in roasted beans was reportedly 12% higher than that in beans without thermal treatment [47]. Additionally, Chelladurai and Chinnachamy [48] reported that the IC50 of α-amylase inhibition activity was reached when a 735 µg concentration of an aqueous extract of Salacia oblonga was used; those values are higher than the results obtained in this study. The rachis aqueous extraction process made the α-amylase inhibition activity more efficient due to the high concentrations of phenolic compounds in the extract. It has also been reported that both polyphenols and flavonoids act as natural antidiabetic agents that inhibit digestive enzymes and free radical production, thereby lowering oxidative stress and decreasing postprandial glucose levels [49].

### 2.4. The α-Glucosidase Inhibition Activity

The α-glucosidase inhibition activity by the action of acarbose at concentrations of 0.5 mg/mL, 1 mg/mL and 5 mg/mL was 15.83%, 18.72% and 53.85%, respectively. However, the aqueous extracts and powder samples did not show any α-glucosidase inhibition activity, so we concluded that the antidiabetic activity of *Chamaedorea tepejilote* Liebm is not mediated by this mechanism of action.

Some plant extracts have been reported to have higher α-glucosidase inhibitory activity due to their high tannin content [50]. It has also been reported that the glycosides present in the plant extracts act as a substrate for the α-glucosidase enzyme and may be responsible for the inhibitory effect [51]. It is worth noting that the presence of tannins and glycosides in the rachis extracts was not reported and, probably for this reason, no inhibition of the α-glucosidase enzyme was observed.

### 2.5. The Potential of Rachis as Dipeptidyl Peptidase-IV Inhibitors

As shown in Table 4, the DPP-IV inhibition activity was different according to thermal treatment and dose tested. The rachis extracts that had undergone steaming at an elevated pressure showed the highest inhibition activity at the lowest doses; however, all thermal treatments and doses showed inhibition activity that was higher than 10%.

When rachis powder was used as an inhibitor agent of DPP-IV, low inhibition activity values were obtained (Table 5). The highest inhibition activity was observed in rachis powder samples that had undergone steaming at an elevated pressure. Additionally, inhibition activity was observed in a greater number of doses among rachis powder samples that had undergone wet treatments than dry or no thermal treatment.

The highest DPP-IV inhibition activity was observed in rachis extracts at a dose of 10 μg/mL that had undergone steaming at an elevated pressure; however, at higher doses, the high concentrations of inhibitor components in the rachis samples probably saturated the medium, thereby its inhibition efficiency was reduced. In addition, the non-specific composition of the powder samples generated diverse reactions, which decreased the inhibition of specific enzymes by their components. For this reason, the inhibition activity in the rachis extract samples was higher than the powder. The results obtained were in concordance with those reported by Zeytünlüoğlu and Zihnioğlu [52], who tested the antidiabetic activity of extracts and demonstrated that thermal treatment improved the DPP-IV inhibitory effect of *Vitis vinífera* and *Artemisia dracunculus*.

It has been reported that thermally treated samples increase their enzyme inhibition capacity against the DPP—IV enzyme, e.g., thermally treated samples of *Phaseolus vulgaris* showed higher inhibitory activity than crude *Phaseolus vulgaris* samples [53]. Some authors have attributed the inhibitory effect of the extracts to flavonoids such as cirsimaritin, hispidulin, naringenin, galangin and quercetin [54,55,56]. On the other hand, coumarins have also been reported to be able to inhibit the DPP-IV enzyme [57]. Previous qualitative studies have shown that flavonoids and coumarins are present in the aqueous extracts of rachis and that these compounds may be responsible for the DPP-IV inhibitory activity. For all samples, a Gaussian behavior was observed; for this reason, the DPP-IV inhibition tends to increase at the beginning with low inhibitor concentrations, then decrease with higher inhibitor concentrations after reaching the maximum value.

### 2.6. The Angiotensin-Converting Enzyme (ACE) Inhibition Activity

As can be seen in Table 6, no inhibition of ACE was observed in the extracts obtained from samples subjected to dry thermal treatment or no thermal treatment at the concentrations tested. However, in the two wet thermal treatments (hydrothermal processing and steaming at elevated pressure), the highest inhibitory activity against ACE was only achieved at the higher dose tested.

ACE inhibition activity was only observed in the powder samples that had undergone the hydrothermal processing treatment. The highest ACE inhibition activity in the samples only reached about 10% of the maximal inhibition activity of the captopril. For this reason, the antihypertensive activity of the samples mediated by ACE inhibition was considered to be low, also taking into account the fact that methanolic and aqueous extracts of *Phoenix sylvestris* showed inhibition levels of 35% and 33%, respectively [58].

In the extracts, a higher number of samples showed inhibition of ACE, probably due to the fact that, in the powder, the concentration of antihypertensive compounds is low due to the number of compounds that make up the food matrix. Whereas in the aqueous extracts there are probably more specific compounds such as flavonoids, quinones, phenols and coumarins. Thermal treatments have been reported to increase activity against ACE in some samples. For example, Ahmed et al. [59] reported that hydrothermal treatment of *Ficus racemosa* increased the antihypertensive activity by 100-fold to reach the IC50 value against ACE, while 400 µg/mL and 4 µg/mL were required to achieve 90% ACE inhibition in the fresh sample and 100% in the hydrothermally treated sample, respectively.

### 2.7. Pearson Correlation Analysis

A moderate Pearson correlation was observed between the total phenolic compounds of the rachis extracts and the DPP-IV and α-amylase inhibition activity, with values of r = 0.662 and r = 0.794, respectively. These results were concordant with those reported by Lacroix and Li [60], who indicated that total phenolic compounds and digestive enzymes had positive correlations. Additionally, Saliu et al. [61] indicated that the phenolic compounds extracted from *Vernonia amigdalina* samples had positive correlations with α-amylase inhibition activity. Our obtained results could be a first step toward the characterization of pacaya palm inflorescence rachis as an adjuvant therapy for the treatment of glucose metabolism disorders.

## 3. Materials and Methods

### 3.1. Plant Materials

The pacaya palm was obtained from a local market in Tapachula, Chiapas, Mexico. The pacaya palm was transported to the laboratory in a cardboard box. The bracts of male inflorescences were removed, and the rachis were cut into cubes that were approximately 0.5 cm in length. The rachis were then packed into PVC bags in batches of 300 g and stored at 4 °C in a refrigerator (GR-452SH model, LG electronics, Monterrey, N.L., Mexico) for no more than 24 h. For all thermal treatments, the samples were vacuum packed into PVC bags.

### 3.2. Thermal Treatments

We evaluated three heat treatments involving different heat transfer mechanisms, as described by Hernández et al. [25].

For steaming at an elevated pressure, a 300 g sample of rachis was placed on a tray in a pressure cooker and steamed for 15 min at 125 °C under a pressure of 124,106 Pa. For the hydrothermal processing treatment, a 300 g sample of rachis was placed in a water bath at 90 °C for 15 min. Finally, for the microwave treatment, a 300 g sample of rachis was placed in a 1500 W microwave oven with an operating frequency of 2450 MHz (Goldstar MS-157XC model, LG Corp., Seoul, Republic of Korea) and cooked for 15 min at 90 °C.

All thermally treated samples and a sample without thermal treatment (control) were then cooled to room temperature and frozen at −20 °C for 24 h. They were later lyophilized using a laboratory freeze dryer (Scientz-10N, Ningbo, China).

### 3.3. Preparation of Powder

After the freeze-drying process, all samples were milled into a fine powder and sieved through a size 80 mesh. The powder was packaged in 25 g glass bottles and stored at 25 °C until use.

### 3.4. Preparation of Rachis Extracts

To prepare the rachis extracts, 5 g of the rachis powder was mixed with 100 mL of solvent (water or hexane) in a conical flask. The samples were then shaken with a mechanical shaker (Eberbach, 5900, Ann Arbor, MI, USA) for 24 h at room temperature. The extracts were filtered through Whatman No. 4 filter paper. For the samples with hexane, the solvent was evaporated using a rotary evaporator (DragonLab, RE100-Pro, China), while the aqueous extracts were lyophilized to eliminate any remaining water. The extracts without solvent were stored in a nitrogen atmosphere at 4 °C in the dark until analysis [62]. The yields of the extracts were calculated using Equation (1):

(1) $$Extract\;yield\left(\%\right)=\frac{Final\;weigth}{Initial\;weight}\times 100$$

where Initial weight is the amount of rachis mixed and Final weight is the amount of extract recovered after eliminating the solvent.

### 3.5. Determination of the Total Phenol Content of the Extracts

To establish the total phenolic content, phenolic extraction was carried out according to Cardador et al. [63]. The quantification of the total phenol content was carried out according to Rajha et al. [64], with some modifications. In brief, 200 µg of dry sample was mixed with 1580 µL of water and 100 µL of Folin–Ciocalteu reagent (Folin & Ciocalteu’s phenol reagent, Sigma Aldrich, St. Louis, MO, USA). Then, the mixture was left to stand at room temperature for 8 min and later, 300 µL of 30% sodium carbonate solution was added. The absorbance was measured at 760 nm after 90 min of rest at room temperature using a spectrophotometer (Optima Plus, SP 3000 nano, USA). The results were expressed as the gallic acid equivalent (GAE) in milligrams per gram of extract using a gallic acid (Sigma-Aldrich, St. Louis, MO, USA) standard curve.

### 3.6. Evaluation of α-Amylase Inhibition Activity

The ability of the rachis extract and powder samples to inhibit α-amylase activity was assessed according to the methodology of Nair et al. [65]. Briefly, 200 μL of 0.02 M, pH 6.9 sodium phosphate buffer, 20 μL of the enzyme at a concentration of 1 U/mL (α-amylase from Bacillus licheniformis, Sigma Aldrich Co., EU) and samples (aqueous extract or heat-treated and untreated tepejilote rachis powder) at concentrations of 250–500 μg/mL were incubated for 10 min at room temperature and then 200 μL of 1% soluble starch was added to all test tubes. The reaction was terminated with the addition of 400 μL of DNS (3,5-dinitrosalicylic acid, Sigma Aldrich, St. Louis, MO, USA) reagent and the samples were then placed in a boiling water bath for 5 min, cooled and diluted with 15 mL of distilled water. Absorbance was measured at 540 nm. The negative control was prepared without adding any extract. As a positive control, acarbose samples at concentrations of 250–500 μg/mL were prepared under same conditions. The % inhibition was calculated using Equation (2):

(2) $$\%\;Inhibition=\frac{A-B}{A}*100$$

where A is the absorbance of the negative control (100% enzyme activity) and B is the absorbance of the sample. 

### 3.7. Evaluation of α-Glucosidase Inhibition Activity

The evaluation of the α-glucosidase inhibition activity was carried out following the methodology of Zheng et al. [66], with some modifications. Briefly, 560 μL of buffer (sodium phosphate buffer, pH 6.8–6.9, at 0.1 M), 100 μL of α-glucosidase (0.4 U/mL in a sodium phosphate buffer, pH 6.8–6.9, at 0.1 M) (Sigma Aldrich, St. Louis, MO, USA) and 40 μL of sample (aqueous extract or heat-treated and untreated tepejilote rachis powder) were incubated at 37 °C for 15 min. Then, 100 μL of the substrate p-nitrophenyl-α-glucopyranoside (5 mM, Sigma Aldrich, St. Louis, MO, USA) (reconstituted in the sodium phosphate buffer, pH 6.8–6.9, at 0.1 M) was added and the samples were incubated again at 37 °C for 30 min. The enzymatic reaction was terminated by adding sodium carbonate (0.2 M). The absorbance was determined at 405 nm using a UV-VIS spectrophotometer (Optima Plus, SP 3000 nano, USA). Acarbose at concentrations of 0.5 mg/mL, 1 mg/mL and 5 mg/mL (Sigma Aldrich, St. Louis, MO, USA) was used as the positive control and the negative control received no treatment. The % of inhibition was calculated using Equation (3):

(3) $$Inhibition\;\left(\%\right)=\frac{1-\left(A-B\right)}{\left(C-B\right)}*100$$

where A is the absorbance of the sample (sample, enzyme and substrate), B is the absorbance of the blank (buffer only) and C is the absorbance of the negative control (enzyme and substrate without a sample). For the positive control, acarbose was used instead of a sample and was considered as A.

### 3.8. Evaluation of Dipeptidyl Peptidase-IV (DPP-IV) Inhibition Activity

The evaluation of the dipeptidyl peptidase-IV (DPP-IV) inhibition activity was carried out based on the methodology of Lin et al. [67], with some modifications. In brief, 50 µL of the test sample (aqueous extract or heat-treated and untreated tepejilote rachis powder) at different concentrations (10–600 μg/mL) was mixed with 50 μL of DPP-IV (Sigma Aldrich, Co., St. Louis, MO, USA) (previously reconstituted (0.002 U/mL) in a Tris buffer, pH 8, at 0.1 M0). The mixture was then incubated at 37 °C for 10 min. Next, 50 μL of the substrate Gly-Pro p-nitroanilide (Sigma Aldrich, Co., St. Louis, MO, USA) (1 mM reconstituted in the Tris buffer, pH 8 at 0.1 M) and 50 μL of the Tris buffer (pH 8, at 0.1 M) were added and incubated at 37 °C for 60 min. Then, 50 μL of 3% acetic acid was added to stop the reaction. The absorbance was measured at 405 nm using a spectrophotometer (Optima Plus, SP 3000 nano, USA). Sitagliptin (Merck Sharp & Dohme, Rahway, NJ, USA) at a concentration of 0.1 μM was used as the positive control. The sample background was each sample with the Tris buffer only. The % inhibition was calculated using Equation (4):

(4) $$DPP-IV\;Inhibition\left(\%\right)=\frac{A-\left(B-C\right)}{\left(A\right)}*100$$

where A is the absorbance of the negative control (enzyme and substrate), B is the absorbance of the sample (sample, enzyme and substrate) and C is the absorbance of the background (sample and buffer).

### 3.9. Evaluation of Angiotensin-Converting Enzyme (ACE) Inhibition Activity

The ACE inhibition activity was determined using the method of Chaudhary et al. [68]. In brief, 50 μL of sample (aqueous extract or heat-treated and untreated tepejilote rachis powder) and 50 μL of ACE solution (Sigma-Aldrich, A2580) (25 mU/mL) were preincubated at 37 °C for 10 min. Then, the mixture was incubated with 150 μL of substrate (8.3 mM n-hypuryl-histidyl-leucine, 0.3% *w*/*v*; 50 mM of borate buffer with 0.3 M NaCl, pH 8.3, reconstituted in 50 mM of sodium borate buffer containing 0.3 M NaCl, pH 8.3) for 30 min at the same temperature. The reaction was terminated by the addition of 250 μL of 1.0 M HCl. The resulting hippuric acid was extracted using 0.5 mL of ethyl acetate and centrifuged using a Hermle Z323K centrifuge (Hermle, Laborthechnic Germany) for 15 min at 1000 rpm. Then, 200 μL of the upper layer was transferred into a test tube and evaporated at room temperature for 2 h in a vacuum. The hippuric acid was dissolved in 1.0 mL of distilled water and the absorbance was measured at 228 nm using a UV spectrophotometer (Optima Plus, SP 3000 nano, USA). The % inhibition was calculated using Equation (5):

(5) $$ACE\;Inhibition\;\left(\%\right)=\frac{\left(A-C\right)}{\left(A-B\right)}*100$$

where A is the absorbance of the negative control (substrate and enzyme), B is the absorbance of the blank (buffer only) and C is the absorbance of the sample (enzyme, substrate and sample). For the positive control, captopril was used instead of a sample and was considered as C.

### 3.10. Experimental Design and Statistical Analysis

A randomized 2 × 4 factorial design was used for the extraction method. Two solvents were considered for the extraction factor (hexane and water) and four levels were used for the thermal treatment factor (without thermal treatment, hydrothermal treatment, boiling in the microwave and steam pressure). A randomized 2 × 4 factorial design was used for the enzymatic test. The samples tested were aqueous extract and powder with the thermal treatment factor at the four levels of thermal treatment. The response variables considered were total phenolic and enzymatic activity (α-amylase, α-glucosidase and ACE).

The quantitative data were expressed as the means ± standard deviation and an analysis of variance (ANOVA) was performed, followed by Tukey’s test. SAS software was used for the data analysis and all experimental determinations were performed in triplicate.

The Pearson correlation coefficient (r) was assessed to determine the correlations between total phenolic compound (TPC) content and the inhibitory effects of α-amylase and dipeptidyl peptidase-IV (DPP-IV). Correlations were determined to be significant at *p* < 0.05. The Origin Pro, Version 2021 (OriginLab Corporation, Northampton, MA, USA) statistical package was used.

## 4. Conclusions

For the extraction of nutraceutical components from the rachis, water is the better extractant than hexane, as a four-fold yield was obtained in some samples when water was used as the extractant. On the other hand, a more efficient extraction of phenolic compounds was observed with a wet process than with a dry process. The rachis of pacaya shows an inhibitory effect on the enzymes α-amylase and DPP-IV, which are involved in glucose metabolism and, for this reason, may then contribute to its blood glucose-lowering effect. Thermal treatment increases the inhibition of α-amylase, also thermal treatment with steam under pressure allowed the greatest release of inhibitory components against DPP-IV, as it showed a percentage inhibition of over 50% at low doses. On the other hand, no inhibitory effect was observed against α-glucosidase, so rachis does not seem to be involved in this pathway of glucose metabolism, apparently due to the fact that extracts of tepejilote rachis do not contain inhibitory compounds against this enzyme. A slight ACE inhibitory activity was observed, so that the rachis of the pacaya palm could be used as a supportive therapy in the treatment of hypertension, especially the extracts obtained from the rachis subjected to a wet thermal treatment. The results obtained show that not only the extraction yield but also the extracted properties were modified by the extraction method and the temperature used.

Significant positive correlations were found between TPC content and α-amylase and DPP-IV inhibitory activity, supporting the idea that the phenolic compounds in the plants are potent inhibitors of the enzymes involved in glucose metabolism.

## Figures and Tables

**Table 1:** Percentage yields of pacaya palm inflorescence rachis extracts after different thermal treatments.

Treatment	Yield (%)	
	Water	Hexane
Without thermal treatment	10.51 ^b^ ± 0.24	2.77 ^d^ ± 0.14
Microwaving	10.85 ^b^ ± 0.24	4.10 ^c^ ± 0.15
Hydrothermal processing	17.08 ^a^ ± 1.56	4.51 ^b,c^ ± 0.18
Steaming at elevated pressure	17.88 ^a^ ± 0.54	6.44 ^a^ ± 0.20

Values in the same column followed by different lowercase superscript letters differed significantly (*p* < 0.05).

**Table 2:** Total phenolic content in the rachis extracts after different thermal treatments.

Treatment	Total Phenolic Content (μg EAG/g of Extract)	
	Water	Hexane
Without thermal treatment	14340.17 ^d^ ± 38.52	197.71 ^a^ ± 22.21
Microwaving	14551.45 ^c^ ± 47.18	330.04 ^b^ ± 42.50
Hydrothermal processing	14851.68 ^a^ ± 94.36	629.16 ^c^ ± 10.00
Steaming at elevated pressure	14651.53 ^b^ ± 66.72	660.30 ^c^ ± 29.15

Values in the same column followed by different lowercase superscript letters differed significantly (*p* < 0.05).

**Table 3:** Inhibition of α-amylase in rachis aqueous extracts and powder.

Treatment	Inhibition of α-Amylase (%)			
	Concentration of Extract/Acarbose			
	250 μg/mL		500 μg/mL	
	Powder	Aqueous Extract	Powder	Aqueous Extract
Positive control (Acarbose)	$44.00\;\begin{array}{c} b\\ B \end{array}$ ± 4.00	$44.00\;\begin{array}{c} b\\ B \end{array}$ ± 4.00	$73.33\;\begin{array}{c} a\\ A \end{array}$ ± 2.31	$73.33\;\begin{array}{c} b\\ A \end{array}$ ± 2.31
Without thermal treatment	$26.00\;\begin{array}{c} c\\ C \end{array}$ ± 2.83	$38.67\;\begin{array}{c} c\\ B \end{array}$ ± 2.31	$38.00\;\begin{array}{c} e\\ B \end{array}$ ± 2.83	$72.00\;\begin{array}{c} b\\ A \end{array}$ ± 4.00
Microwaving	$49.33\;\begin{array}{c} a,b\\ B \end{array}$ ± 2.31	$54.00\;\begin{array}{c} a,b\\ B \end{array}$ ± 2.83	$54.67\;\begin{array}{c} d\\ B \end{array}$ ± 2.31	$70.00\;\begin{array}{c} b\\ A \end{array}$ ± 2.83
Hydrothermal processing	$56.00\;\begin{array}{c} a\\ C \end{array}$ ± 5.66	$57.33\;\begin{array}{c} a\\ B,C \end{array}$ ± 2.31	$66.00\;\begin{array}{c} c\\ B \end{array}$ ± 2.83	$85.33\;\begin{array}{c} a\\ A \end{array}$ ± 2.31
Steaming at elevated pressure	$46.67\;\begin{array}{c} b\\ C \end{array}$ ± 2.31	$52.00\;\begin{array}{c} a,b\\ C \end{array}$ ± 5.66	$69.33\;\begin{array}{c} b,c\\ B \end{array}$ ± 2.31	$81.33\;\begin{array}{c} a,b\\ A \end{array}$ ± 2.31

Values in the same column followed by different lowercase superscript letters differed significantly (*p* < 0.05); values in the same row followed by different uppercase subscript letters differed significantly (*p* < 0.05); acarbose was used as a pharmacology agent inhibitor.

**Table 4:** Inhibition of dipeptidyl peptidase-IV (DPP-IV) by the rachis extracts.

Treatment	Inhibition of DPP-IV (%)				IC50 (μg/mL)
	Concentration of Extract				
	10 μg/mL	50 μg/mL	500 μg/mL	600 μg/mL	
Without thermal treatment	$28.95\;\begin{array}{c} b\\ C \end{array}$ ± 1.14	$33.00\;\begin{array}{c} b\\ B \end{array}$ ± 0.16	$64.62\;\begin{array}{c} a\\ A \end{array}$ ± 0.12	$29.50\;\begin{array}{c} c\\ C \end{array}$ ± 0.16	496.66
Microwaving	$21.78\;\begin{array}{c} c\\ C \end{array}$ ± 1.27	$40.42\;\begin{array}{c} a\\ A \end{array}$ ± 0.13	$23.17\;\begin{array}{c} c\\ C \end{array}$ ± 0.92	$29.82\;\begin{array}{c} c\\ B \end{array}$ ± 0.31	20.83
Hydrothermal processing	$14.99\;\begin{array}{c} d\\ B \end{array}$ ± 1.55	$14.18\;\begin{array}{c} d\\ B \end{array}$ ± 0.13	$14.04\;\begin{array}{c} d\\ B \end{array}$ ± 0.38	$37.43\;\begin{array}{c} a\\ A \end{array}$ ± 0.13	622.64
Steaming at elevated pressure	$62.57\;\begin{array}{c} a\\ A \end{array}$ ± 0.12	$23.68\;\begin{array}{c} c\\ C \end{array}$ ± 0.44	$30.48\;\begin{array}{c} b\\ B \end{array}$ ± 0.22	$31.94\;\begin{array}{c} b\\ B \end{array}$ ± 0.13	6.12

Values in the same column followed by different lowercase superscript letters differed significantly (*p* < 0.05); values in the same row followed by different uppercase subscript letters differed significantly (*p* < 0.05).

**Table 5:** Inhibition of DPP-IV by the rachis powder.

Treatment	Inhibition of DPP-IV (%)			
	Concentration of Extract			
	10 μg/mL	50 μg/mL	500 μg/mL	600 μg/mL
Without thermal treatment	n.d.	$4.86\;\begin{array}{c} b\\ B \end{array}$ ± 0.19	$10.17\;\begin{array}{c} a\\ A \end{array}$ ± 0.26	n.d.
Microwaving	n.d.	$8.05\;\begin{array}{c} a\\ A \end{array}$ ± 0.15	n.d.	n.d.
Hydrothermal Processing	$3.05\;\begin{array}{c} b\\ A \end{array}\;$± 0.27	n.d.	$4.07\;\begin{array}{c} c\\ A \end{array}$ ± 0.22	$1.49\;\begin{array}{c} a\\ B \end{array}$ ± 0.15
Steaming at Elevated Pressure	$12.85\;\begin{array}{c} a\\ A \end{array}$ ± 0.22	$2.49\;\begin{array}{c} c\\ B \end{array}$ ± 0.19	$15.78\;\begin{array}{c} b\\ A \end{array}$ ± 0.22	n.d.

Values in the same column followed by different lowercase superscript letters differed significantly (*p* < 0.05); values in the same row followed by different uppercase subscript letters differed significantly (*p* < 0.05); n.d., not detected at this concentration.

**Table 6:** The inhibition of angiotensin-converting enzyme (ACE) in rachis aqueous extracts and powder.

Sample	Treatment	Inhibition of ACE (%)				
		Concentration of Extract/Captopril				
		160 μg/mL	240 μg/mL	320 μg/mL	400 μg/mL	500 μg/mL
Captopril (Positive Control)		91.29 $\begin{array}{c} a\\ A \end{array}$ ± 0.66	93.18 $\begin{array}{c} a\\ A \end{array}$ ± 1.14	95.08 $\begin{array}{c} a\\ A \end{array}$ ± 0.66	96.59 $\begin{array}{c} a\\ A \end{array}$ ± 1.14	97.35 $\begin{array}{c} a\\ A \end{array}$ ± 1.14
Extracts	No treatment	n.d.	n.d.	n.d.	n.d.	n.d.
	Microwaving	n.d.	n.d.	n.d.	n.d.	n.d.
	Hydrothermal processing	1.70 $\begin{array}{c} c\\ C \end{array}$ ± 0.80	4.55 $\begin{array}{c} b\\ B \end{array}$ ± 1.61	5.11 $\begin{array}{c} b,c\\ B \end{array}$ ± 0.80	7.95 $\begin{array}{c} b\\ A \end{array}$ ± 1.61	8.52 $\begin{array}{c} b\\ A \end{array}$ ± 0.80
	Steaming at elevated pressure	3.03 $\begin{array}{c} b\\ B \end{array}$ ± 0.66	4.17 $\begin{array}{c} b\\ B \end{array}$ ± 1.14	7.19 $\begin{array}{c} b\\ A \end{array}$ ± 0.66	8.33 $\begin{array}{c} b\\ A \end{array}$ ± 1.31	8.71 $\begin{array}{c} b\\ A \end{array}$ ± 0.66
Powder	No treatment	n.d.	n.d.	n.d.	n.d.	n.d.
	Microwaving	n.d.	n.d.	n.d.	n.d.	n.d.
	Hydrothermal processing	n.d.	5.68 $\begin{array}{c} b\\ C \end{array}$ ± 1.97	4.17 $\begin{array}{c} c\\ C \end{array}$ ± 1.74	7.58 $\begin{array}{c} b\\ B \end{array}$ ± 1.74	9.09 $\begin{array}{c} b\\ A \end{array}$ ± 1.97
	Steaming at elevated pressure	n.d.	n.d.	n.d.	n.d.	n.d.

Values in the same column followed by different lowercase superscript letters differed significantly (*p* < 0.05); values in the same row followed by different uppercase subscript letters differed significantly (*p* < 0.05); n.d., not detected at this concentration; captopril was used as a pharmacology agent inhibitor.

## Data Availability

Data are contained within the article.

## References

[1] Adefegha S.A. (2017). Functional Foods and Nutraceuticals as Dietary Intervention in Chronic Diseases; Novel Perspectives for Health Promotion and Disease Prevention. J. Diet. Suppl..

[2] Mukhtar Y., Galalain A., Yunusa U. (2020). A modern overview on diabetes mellitus: A chronic endocrine disorder. Eur. J. Biol. Sci..

[3] Tong D.P., Zhu K.X., Guo X.N., Peng W., Zhou H.M. (2018). The enhanced inhibition of water extract of black tea under baking treatment on α-amylase and α-glucosidase. Int. J. Biol. Macromol..

[4] Krentz A.J., Bailey C.J. (2005). Oral Antidiabetic Agents. Drugs.

[5] Tseng P.S., Ande C., Moremen K.W., Crich D. (2023). Influence of side chain conformation on the activity of glycosidase inhibitors. Angew. Chem..

[6] Compain P., Martin R.O. (2007). Iminoasugars: Past, Presents and Future.

[7] Rajasekaran P., Ande C., Vankar Y.D. (2022). Synthesis of (5, 6 & 6, 6)-oxa-oxa annulated sugars as glycosidase inhibitors from 2-formyl galactal using iodocyclization as a key step. Arkivoc.

[8] Menzel M., Ziegler T. (2014). Synthesis of vicinal diketoses by using a metathesis–hydroxylation–oxidation sequence. Eur. J. Org. Chem..

[9] Gallwitz B. (2008). Dipeptidyl Peptidase-4 Inhibitors in Type 2 Diabetes Therapy. Eur. J. Endocrinol..

[10] Nauck M.A. (2011). Incretin-Based Therapies for Type 2 Diabetes Mellitus: Properties, Functions, and Clinical Implications. Am. J. Med..

[11] Conarello S.L., Li Z., Ronan J., Roy R.S., Zhu L., Jiang G., Liu F., Woods J., Zycband E., Moller D.E. (2003). Mice lacking dipeptidyl peptidase IV are protected against obesity and insulin resistance. Proc. Natl. Acad. Sci. USA.

[12] Mittal B.V., Singh A.K. (2010). Hypertension in the Developing World: Challenges and Opportunities. Am. J. Kidney Dis..

[13] Verma T., Sinha M., Bansal N., Yadav S.R., Shah K., Chauhan N.S. (2020). Plants Used as Antihypertensive. Nat. Prod. Bioprospect..

[14] Guang C., Phillips R.D. (2009). Plant Food-Derived Angiotensin I Converting Enzyme Inhibitory Peptides. J. Agric. Food Chem..

[15] Chaudhary N., Sabikhi L., Hussain S.A., MH S.K. (2020). A comparative study of the antioxidant and ACE inhibitory activities of selected herbal extracts. J. Herb. Med..

[16] Tadera K., Minami Y., Takamatsu K., Matsuoka T. (2006). Inhibition of α-glucosidase and α-amylase by flavonoids. J. Nutr. Sci. Vitaminol..

[17] Bnouham M.M., Ziyyat A.A., Mekhfi H.H., Tahri A.A., Legssyer A.A. (2006). Medicinal plants with potential antidiabetic activity—A review of ten years of herbal medicine research (1990–2000). J. Diabetes Metab..

[18] Aadil K.R., Barapatre A., Rathore N., Pottam S., Jha H. (2012). Comparative study of in-vitro antioxidant and antidiabetic activity of plant extracts of Acacia arabica, Murraya koeingii, Catharanthus roseus and Rouwolfia serpentina. Int. J. Phytomedicine..

[19] Yarizade A., Kumleh H.H., Niazi A.L.I. (2017). In vitro antidiabetic effects of ferula assa-foetida extracts through dipeptidyl peptidase iv and α-glucosidase inhibitory activity. Asian J. Pharm. Clin. Res..

[20] Jiménez-Arellanes A., Luna-Herrera J., Cornejo-Garrido J., López-García S., Castro-Mussot M.E., Meckes-Fischer M., Mata-Espinosa D., Marquina B., Torres J., Hernández-Pando R. (2013). Ursolic and oleanolic acids as antimicrobial and inmunomodulatory compounds for tuberculosis treatment. BMC Complement. Altern. Med..

[21] Pérez G.C., Zavala S.M.A., Ventura R.E., Pérez G.S., Ponce M.H. (2008). Evaluation of anti-tussive activity of *Chamaedorea tepejilote*. J. Ethnopharmacol..

[22] Jiménez A., Meckes M., Alvarez V., Torres J., Parra R. (2005). Secondary metabolites from *Chamaedora tepejilote* (Palmae) are active against Mycobacterium tuberculosis. Phytother. Res..

[23] Riquett Robles D.J., Solórzano Carranza E.R. (2013). Hypoglycemic activity of *Chamaedorea tepejilote* Liebm. (pacaya). Rev. Cubana. Plantas Med..

[24] Mancera-Castro P., Bernardino-Nicanor A., Juárez-Goiz J.M.S., Teniente-Martínez G., González-Cruz L. (2022). Effect of the Type of Thermal Treatment on the Nutritional and Nutraceutical Characteristics of Pacaya Inflorescences (Chamaedorea tepejilote Liebm). Biol. Life Sci. Forum..

[25] Hernández-Castillo J.B.E., Bernardino-Nicanor A., Vivar-Vera M.d.l.Á., Montañez-Soto J.L., Teniente-Martínez G., Juárez-Goiz J.M.S., González-Cruz L. (2020). Modifications of the Protein Characteristics of Pacaya Caused by Thermal Treatment: A Spectroscopic, Electrophoretic and Morphological Study. Polymers.

[26] Jaramillo-Flores M.E., González-Cruz L., Cornejo-Mazon M., Dorantes-Alvarez L., Gutierrez-Lopez G.F., Hernandez-Sanchez H. (2003). Effect of thermal treatment on the antioxidante activity and content of carotenoids and phenolic compounds of cactus pear cladodes (*Opuntia ficus-indica*). Food Sci. Technol. Intl..

[27] Kadja A.B., Atsain-Allangba R.M., Kouadio K.B., Mamyrbékova-Békro A.J., Yves-Alain B. (2020). Influence of temperature on the phytochemical composition and the antioxidant and anticariogenic activities of extracts from the husk of the fruit of *Cocos nucifera* L. (Arecaceae). GSC Biol. Pharm. Sci..

[28] Wang T., He F., Chen G. (2014). Improving bioaccessibility and bioavailability of phenolic compounds in cereal grains through processing technologies: A concise review. J. Funct. Foods.

[29] Gao Y., Xia W., Shao P., Wu W., Chen H., Fang X., Mu H., Xiao J., Gao H. (2022). Impact of thermal processing on dietary flavonoids. Curr. Opin. Food Sci..

[30] Osoś A., Jankowska P., Drozdzynska A., Rózanska M.B., Bieganska-Marecik R., Baranowska H.M., Ruzkowska M., Kacainova M., Tomkowiak A., Kieliszek M. (2022). Pasta with kiwiberry (*Actinidia arguta*): Effect on structure, quality, consumer acceptance, and changes in bioactivity during thermal treatment. Foods.

[31] Kowalczewski P.L., Olejnik A., Bialas W., Kubiak P., Siger A., Nowicki M., Lewandowicz G. (2019). Effect of thermal processing on antioxidant activity and cytotoxicity of waste potato juice. Open Life Sci..

[32] Hsieh H.J., Lin J.A., Chen K.T., Cheng K.C., Hsieh C.W. (2021). Thermal treatment enhances the α-glucosidase inhibitory activity of bitter melon (*Momordica charantia*) by increasing the free form of phenolic compounds and the contents of Maillard reaction products. J. Food Sci..

[33] Pina-Pérez M.C., Rivas A., Martínez A., Rodrigo D. (2018). Effect of thermal treatment, microwave, and pulsed electric field processing on the antimicrobial potential of açaí (*Euterpe oleracea*), stevia (*Stevia rebaudiana Bertoni*), and ginseng (*Panax quinquefolius L*.) extracts. Food Control.

[34] El Gueder D., Maatouk M., Kalboussi Z., Daouefi Z., Chaaban H., Ioannou I., Luis J. (2018). Heat processing effect of luteolin on anti-metastasis activity of human glioblastoma cells U87. Environ. Sci. Pollut. Res..

[35] Roy M.K., Takenaka M., Isobe S., Tsushida T. (2007). Antioxidant potential, anti-proliferative activities, and phenolic content in water-soluble fractions of some commonly consumed vegetables: Effects of thermal treatment. Food Chem..

[36] Dhawan D., Gupta J. (2016). Comparison of Different Solvents for Phytochemical Extraction Potential from *Datura metel* Plant Leaves. Int. J. Biol. Chem..

[37] Uc-Cachón A.H., Dzul-Beh A.D.J., Palma-Pech G.A., Jiménez-Delgadillo B., Flores-Guido J.S., Gracida-Osorno C., Molina-Salinas G.M. (2021). Antibacterial and antibiofilm activities of Mayan medicinal plants against Methicillin-susceptible and -resistant strains of *Staphylococcus aureus*. J. Ethnopharmacol..

[38] Esteves B., Graca J., Pereira H. (2008). Extractive composition and summative chemical analysis of thermally treated eucalypt Wood. Holzforschung.

[39] Lambri M., Torchio F., Colangelo D., Segade S.R., Giacosa S., De Faveri D.M., Rolle L. (2015). Influence of different berry thermal treatment conditions, grape anthocyanin profile, and skin hardness on the extraction of anthocyanin compounds in the colored grape juice production. Food Res. Int..

[40] Huang Y.C., Chang Y.H., Shao Y.Y. (2006). Effects of genotype and treatment on the antioxidant activity of sweet potato in Taiwan. Food Chem..

[41] Choi J.S., Kim H.Y., Seo W.T., Lee J.H., Cho K.M. (2012). Roasting enhances antioxidant effect of bitter melon (Momordica charantia L.) increasing in flavan-3-ol and phenolic acid contents. Food Sci. Biotechnol..

[42] Chumyam A., Whangchai K., Jungklang J., Faiyue B., Saengnil K. (2013). Effects of heat treatments on antioxidant capacity and total phenolic cont ent of four cultivars of purple skin eggplants. Sci. Asia.

[43] Day L., Xu M., Øiseth S.K., Hemar Y., Lundin L. (2010). Control of Morphological and Rheological Properties of Carrot Cell Wall Particle Dispersions through Processing. Food Bioprocess. Technol..

[44] De Roeck A., Sila D.N., Duvetter T., Van Loey A., Hendrickx M. (2008). Effect of high pressure/high temperature processing on cell wall pectic substances in relation to firmness of carrot tissue. Food Chem..

[45] Santos A.F., Argolo A.C., Paiva P.M., Coelho L.C. (2012). Antioxidant activity of *Moringa oleifera* tissue extracts. Phytother. Res..

[46] Bouhafsoun A., Yilmaz M.A., Boukeloua A., Temel H., HARCHE M.K. (2018). Simultaneous quantification of phenolic acids and flavonoids in *Chamaerops humilis* L. using LC–ESI-MS/MS. Food Sci..

[47] Moussou N., Corzo-Martínez M., Sanz M.L., Zaidi F., Montilla A., Villamiel M. (2016). Assessment of Maillard reaction evolution, prebiotic carbohydrates, antioxidant activity and α-amylase inhibition in pulse flours. J. Food Sci. Technol..

[48] Chelladurai G.R.M., Chinnachamy C. (2018). Alpha amylase and alpha glucosidase inhibitory effects of aqueous stem extract of *Salacia oblonga* and its GC-MS analysis. Braz. J. Pharm. Sci..

[49] Kamtekar S., Keer V., Patil V. (2014). Estimation of Phenolic content, Flavonoid content, Antioxidant and Alpha amylase Inhibitory Activity of Marketed Polyherbal Formulation. J. Appl. Pharm. Sci..

[50] Kim J.S., Yang J., Kim M.J. (2011). Alpha glucosidase inhibitory effect, anti-microbial activity and UPLC analysis of *Rhus verniciflua* under various extract conditions. J. Med. Plant Res..

[51] Elya B., Basah K., Mun’Im A., Yuliastuti W., Bangun A., Septiana E.K. (2012). Screening of α-glucosidase inhibitory activity from some plants of *Apocynaceae, Clusiaceae*, Euphorbiaceae, and Rubiaceae. J. Biomed. Biotechnol..

[52] Zeytünlüoğlu A., Zihnioğlu F. (2015). Evaluation of some plants for potential dipeptidyl peptidase IV inhibitory effects in vitro. Turkish J. Biochem..

[53] Mojica L., Chen K., de Mejía E.G. (2014). Impact of Commercial Precooking of Common Bean (*Phaseolus vulgaris*) on the Generation of Peptides, After Pepsin-Pancreatin Hydrolysis, Capable to Inhibit Dipeptidyl Peptidase-IV. J. Food Sci..

[54] Bower A.M., Real Hernandez L.M., Berhow M.A., de Mejia E.G. (2014). Bioactive Compounds from Culinary Herbs Inhibit a Molecular Target for Type 2 Diabetes Management, Dipeptidyl Peptidase IV. J. Agric. Food Chem..

[55] Kalhotra P., Chittepu V., Osorio-Revilla G., Gallardo-Velázquez T. (2019). Discovery of Galangin as a Potential DPP-4 Inhibitor That Improves Insulin-Stimulated Skeletal Muscle Glucose Uptake: A Combinational Therapy for Diabetes. Int. J. Mol. Sci..

[56] Singh A.K., Patel P.K., Choudhary K., Joshi J., Yadav D., Jin J.O. (2020). Quercetin and coumarin inhibit dipeptidyl peptidase-IV and exhibits antioxidant properties: In silico, in vitro, ex vivo. Biomolecules.

[57] Huang P.K., Lin S.R., Chang C.H., Tsai M.J., Lee D.N., Weng C.F. (2019). Natural phenolic compounds potentiate hypoglycemia via inhibition of Dipeptidyl peptidase IV. Sci. Rep..

[58] Jain P.K., Jain S., Sharma S., Paliwal S., Singh G. (2021). Evaluation of anti-diabetic and antihypertensive activity of *Phoenix sylvestris* (L.) Roxb leaves extract and quantification of biomarker Quercetin by HPTLC. Phytomedicine Plus.

[59] Ahmed F., Siddesha J.M., Urooj A., Vishwanath B.S. (2010). Radical scavenging and angiotensin converting enzyme inhibitory activities of standardized extracts of *Ficus racemosa* stem bark. Phytother. Res..

[60] Lacroix I.M., Li-Chan E.C. (2014). Overview of food products and dietary constituents with antidiabetic properties and their putative mechanisms of action: A natural approach to complement pharmacotherapy in the management of diabetes. Mol. Nutr. Food Res..

[61] Saliu J.A., Ademiluyi A.O., Akinyemi A.J., Oboh G. (2012). In vitro antidiabetes and antihypertension properties of phenolic extracts from bitter leaf (*Vernonia amygdalina* Del.). J. Food Biochem..

[62] López-Pantoja Y., Angulo-Escalante M., Martínez-Rodríguez C., Soto-Beltrán J., Chaidez-Quiroz C. (2007). Efecto antimicrobiano de extractos crudos de Neem (*Azadirachta indica* A. Juss) y venadillo (*Swietenia humilis Zucc*) contra E. coli, S. aureus y el bacteriófago P22. Bioquimica.

[63] Cardador-Martínez A., Jiménez-Martínez C., Sandoval G. (2011). Revalorization of cactus pear (*Opuntia* spp.) wastes as a source of antioxidants. Food Sci. Technol..

[64] Rajha H.N., El Darra N., Hobaika Z., Boussetta N., Vorobiev E., Maroun R.G., Louka N. (2014). Extraction of total phenolic compounds, flavonoids, anthocyanins and tannins from grape byproducts by response surface methodology. Influence of solid-liquid ratio, particle size, time, temperature and solvent mixtures on the optimization process. Food Sci. Nutr..

[65] Nair S.S., Kavrekar V., Mishra A. (2013). In vitro studies on alpha amylase and alpha glucosidase inhibitory activities of selected plant extracts. Eur. J. Exp. Biol..

[66] Zheng M., Lu S., Xing J. (2021). Enhanced antioxidant, anti-inflammatory and α-glucosidase inhibitory activities of citrus hesperidin by acid-catalyzed hydrolysis. Food Chem..

[67] Lin Y.S., Chen C.R., Wu W.H., Wen C.L., Chang C.I., Hou W.C. (2015). Anti-α-glucosidase and Anti-dipeptidyl Peptidase-IV Activities of Extracts and Purified Compounds from *Vitis thunbergii* var. taiwaniana. J. Agric. Food Chem..

[68] Chaudhary S.K., Mukherjee P.K., Maiti N., De Kumar A., Bhadra S., Saha B.P. (2013). Evaluation of angiotensin converting enzyme inhibition and anti-oxidant activity of *Piper longum* L.. Indian. J. Tradit. Knowl..

